# IUC24394-87 Meet-URO score update in metastatic renal cell carcinoma receiving first-line immune-combinations

**DOI:** 10.1093/oncolo/oyaf248.041

**Published:** 2025-08-29

**Authors:** Pasquale Rescigno, Aruni Ghose, Nicholas Brown, Lijo James, Sophia Haywood, Ricky Frazer, Anupama Vijay, Mark Stares, Michael Cheung, Ishika Mahajan, Niall Moon, Ritika Abrol, Ondrej Fiala, Vishwani Chauhan, Yamin Shwe Yee Soe, Anum Zargham, Sara Elena Rebuzzi

**Affiliations:** Centre for Cancer, Translational and Clinical Research Institute, Newcastle University, Newcastle Upon Tyne, United Kingdom; Barts Cancer Centre, Barts Health NHS Trust, London, United Kingdom; Cancer Centre at Guy’s, Guy’s and St Thomas’ NHS Foundation Trust, London, United Kingdom; Beatson West of Scotland Cancer Centre, NHS Greater Glasgow and Clyde, Glasgow, United Kingdom; Department of Medical Oncology, The Royal Marsden NHS Foundation Trust, London, United Kingdom; Velindre Cancer Centre, Velindre University NHS Trust, Cardiff, United Kingdom; Leicester Cancer Research Centre, University Hospitals of Leicester NHS Trust, Leicester, United Kingdom; Edinburgh Cancer Centre, NHS Lothian, Edinburgh, United Kingdom; Department of Oncology, Cambridge University Hospitals NHS Foundation Trust, United Kingdom; Lincoln Oncology Centre, United Lincolnshire Hospitals NHS Trust, Lincoln, United Kingdom; Sunrise Oncology Centre, Royal Cornwall Hospitals NHS Trust, Cornwall, United Kingdom; Deanesly Centre for Cancer Services, The Royal Wolverhampton NHS Trust, Wolverhampton, United Kingdom; Department of Oncology and Radiotherapeutics, Faculty of Medicine, University Hospital in Pilsen, Charles University, Prague, Czech Republic; Barts Cancer Centre, Barts Health NHS Trust, London, United Kingdom; Royal Surrey Cancer Centre, Royal Surrey NHS Foundation Trust, Guildford, United Kingdom; Department of Oncology, University Hospitals Birmingham NHS Foundation Trust, Birmingham, United Kingdom; Medical Oncology Unit 2, Molinette Hospital—AOU Città della Salute e della Scienza, Turin, Italy

## Abstract

**Background:**

Risk stratification is fundamental in guiding treatment decisions for mRCC patients. The Meet-URO score is a novel prognostic model (IMDC score + NLR + Bone metastases, PMID 36493602) developed in the immunotherapy era. This innovative score enhanced the prognostic discrimination compared with the IMDC score in patients receiving different therapeutic strategies in different settings.

**Methods:**

A previous external validation on a retrospective real-world cohort of 1174 mRCC patients receiving first-line immune-combinations from 27 centers showed the higher prognostic accuracy of the Meet-URO score compared with the IMDC score (Abstract n. IUC20761-50). Here, we report the updated OS assessment with an exploratory analysis for PFS and ORR.

**Results: A total of:**

1,418 patients from 31 cancer centers were assessed: 54% of patients received IO-IO (Nivolumab + Ipilimumab), 46% IO-TKI, mainly Avelumab + Axitinib (24%); 52.5% of patients had NLR ≥ 3.2, and 32% had bone metastases. After an mFU of 16.5 months, the overall mOS was 34.7 months and resulted in more distinction within the Meet-URO score groups (Table 1), confirming its better prognostic accuracy compared with the IMDC score (c-index: 0.68 vs 0.64), especially for intermediate- and poor-risk patients, while for favorable patients, more FU is needed. Although the Meet-URO score was developed as an OS model, it showed better performance also in terms of PFS (c-index: 0.60 vs 0.58) and ORR (c-index: 0.57 vs 0.54).

**Conclusions:**

The Meet-URO score demonstrated robust and better performance compared with the IMDC score in a large-scale UK mRCC cohort receiving first-line immune-combinations, not only in terms of OS but also in terms of PFS and ORR. The adoption of the Meet-URO score in clinical practice and as a stratification factor of clinical trials could support more individualized patient management.

**Table:** 

**Score**	**Group distribution**	**HR (95% CI)**	***P* value**	**mOS(mo)**	**3y-OS**
** Meet-URO **					
1	13%	1.00 (ref)		45.8	66%
2	27%	1.23 (0.88-1.72)	.23	51.4	62%
3	22%	1.74 (1.24-2.44)	.001	37.6	53%
4	30%	3.31 (2.42-4.51)	<.001	19.7	32%
5	8%	4.82 (3.35-6.94)	<.001	11.5	26%
** IMDC **					
Favorable	19%	1.00 (ref)		45.8	61%
Intermediate	53%	1.40 (1.09-1.79)	.007	37.6	54%
Poor	28%	3.23 (2.51-4.16)	<.001	14.9	30%

